# Preoperatively diagnosed ductal cancers in situ of the breast presenting as even small masses are of high risk for the invasive cancer foci in postoperative specimen

**DOI:** 10.1186/s12957-015-0641-3

**Published:** 2015-07-16

**Authors:** Bartlomiej Szynglarewicz, Piotr Kasprzak, Agnieszka Halon, Rafal Matkowski

**Affiliations:** Department of Surgical Oncology, Lower Silesian Oncology Centre, Plac Hirszfelda 12, 53-413 Wroclaw, Poland; Department of Breast Imaging, Lower Silesian Oncology Centre, Wroclaw, Poland; Department of Pathology, Wroclaw Medical University, Wroclaw, Poland; Department of Oncology, Wroclaw Medical University, Wroclaw, Poland

**Keywords:** Breast cancer, Ductal carcinoma in situ, Core-needle biopsy, Vacuum-assisted biopsy

## Abstract

**Background:**

In ductal carcinoma in situ of the breast (DCIS), histologic diagnosis obtained before the definitive treatment is related to the risk of underestimation if the presence of invasive cancer is found postoperatively. These patients need a second operation to assess the nodal status. We evaluated the upstaging rate in patients with mass-forming DCIS.

**Methods:**

Sixty-three women with pure DCIS presenting as sonographic mass lesion underwent vacuum-assisted or core-needle biopsy and subsequent surgery. Rates of postoperative upstaging to invasive cancer were calculated and compared with clinical character and size of DCIS.

**Results:**

Median age of patients (range) was 63 years (27–88) while median diameter of DCIS was 11 mm (6–60). Fifty-six percent of DCIS were upstaged. Patient age did not differ significantly between groups with and without final invasion (median, mean, SD): 63, 61.4, 12.5 vs 62, 61.2, 10.6 years, respectively (*P* = 0.659). The difference of DCIS size between these groups was statistically important (median, mean, SD): 13, 17.3, 11.4 vs 9.5, 9.8, 3.2 mm, respectively (*P* = 0.0003). Mass size and palpability were significant risk factors (*P* < 0.001 and *P* < 0.01, respectively). Rate of underestimation for mass with diameter ≤10 mm, 10–20 mm and >20 mm was 37, 64 and 91 %, respectively.

**Conclusions:**

DCIS diagnosed on minimal-invasive biopsy of even small sonographic mass is of high risk for the upstaging to invasive cancer after final surgical excision. In these patients, subsequent intervention is needed for nodal status assessment. They are good candidates for the sentinel node biopsy during the breast operation to avoid multi-step surgery.

## Background

The widespread uptake of contemporary breast imaging modalities have resulted in an increase in diagnosis of borderline lesions and preinvasive cancers including ductal carcinoma in situ (DCIS), which is usually presenting on mammography as microcalcifications [1]. The overall natural progression of DCIS to invasive malignancy is reported to range from 14 to 75 %, which reflects their variable malignant potential and biological diversity [2]. Histopathologic diagnosis of breast cancer should be obtained before the definitive treatment using minimal-invasive investigations. However, it is related to the risk of the presence of invasive ductal cancer (IDC) that can be found in postoperative specimen after surgical excision. These patients need subsequent re-operation—usually a sentinel node biopsy to assess the nodal status [3]. Some features of DCIS with more aggressive biology have been identified (high nuclear grade, comedonecrosis), some remain disputable and many others are a matter of investigation [4]. Moreover, not all the variables helpful for optimal treatment planning are available before the final examination of postoperative specimen (e.g. microscopic lesion diameter and surgical margin width covered by Van Nuys Prognostic Index).

Among radiological features, the mammographic extent over than 4–5 cm and the presence of architectural distortion, focal asymmetric density or mass on mammography are proven important risk factors of histological underestimation [5, 6]. In addition, in some reports, a mass lesion visible on ultrasound can be significantly related to the risk of upstaging DCIS to IDC [6]. The cut-off point of lesion size that makes a distinction between low- and high-risk DCIS is still a subject of debate. However, it tends to decrease. Based on recent findings, the significant cut-off point seems to be much smaller than traditional [7].

The primary aim of this study was to assess the postoperative upstaging rate in patients with mass-forming pure DCIS with histologic diagnosis obtained from preoperative, image-guided breast biopsy. The secondary aim was to evaluate its relation to the lesion size, particularly with regard to small, non-palpable DCIS.

## Methods

### Patients

During the years 2004–2014 in our institution, 8403 percutaneous biopsies of suspicious breast masses under ultrasound guidance (vacuum assisted or core needle with automated gun) were performed. In histological examination of biopsy specimens, DCIS was found as a part of targeted mass in 91 patients. Of them, three had breast cancer history: IDC of contralateral breast, DCIS of contralateral breast (diagnosed on stereotactic biopsy of microcalcifications without mass) and IDC of ipsilateral breast (other quadrant), respectively. In every case, mammography and ultrasound were done. Two lesions were presenting as solid mass without any other mammographic manifestation. Mammography demonstrated mass with architectural distortion and mass with focal asymmetric density in three and two patients, respectively. In the remaining cases, DCIS was detected in mammography as a mass with associated microcalcifications, with the highest percentage being crushed stone type, the second-most common linear casting type and the less frequent powderish type. Nevertheless, because the lesions were sonographically visible as evident mass (most common microlobulated, hypoechoic), patients were referred to ultrasound-guided biopsy as the preferable option in our institution in cases of tumours detectable both in mammography and sonography. This management is based on the fact that biopsy under ultrasound guidance has some advantages: it is performed in a real time, direct needle visualization allows accuracy of sampling to be assessed, does not involve ionizing radiation, its sensitivity is higher, procedure is faster and more comfortable for a patient [8–11].

### Pathologic assessment

All haematoxylin-and-eosin-stained slides of formalin-fixed and paraffin-embedded tissue blocks were assessed by board-certified pathologists experienced in breast cancer. In 26 cases, invasion or microinvasion (≤1 mm in the longest diameter) was found—these patients were excluded from the analysis. Then, the subsequent surgical excision was completed. Two women had surgery in other institutions and data from follow-up excision were not available. In the remaining cases, postoperative pathologic examination was performed. Special effort was made to find any foci of IDC in the surgical specimen. All the biopsy and excision slides were reviewed and re-evaluated by study supervising pathologist (AH) to confirm the original diagnosis of DCIS, as well as the absence or presence of invasion or microinvasion. Finally, 63 patients entered the study fulfilling the inclusion criteria: sonographic mass lesion, BIRADS category 4 or 5, ultrasound-guided core needle or vacuum-assisted biopsy, pure DCIS in histologic assessment of biopsy specimens, material of surgical excision available for postoperative pathologic examination.

### Minimally invasive ultrasound-guided biopsy

Ultrasound-guided biopsies were performed with the patients in the supine or supine-oblique position in a breast sonography unit. Biopsies were carried out strictly according to standardized protocol of this procedure by one breast-dedicated radiologist (PK), after reading mammograms and performing diagnostic sonography to individually tailor the biopsy technique. Clinical character of lesion (palpable vs non-palpable) was also examined by the same radiologist. In cases of palpable tumour (29/63), core-needle biopsy with 14-G needle using automated gun was done (Manan Pro-Mag^TM^ 2.2, Angiotech Pharmaceuticals Inc, distributed in Europe by PBN Medicals, Stenlose, Denmark). For non-palpable lesions (34/63), vacuum-assisted biopsy was performed with 11-G needle (Mammotome System MHH 11, Ethicon EndoSurgery Europe, Norderstedt, Germany) or 10-G needle (EnCore Breast Biopsy System, SenoRx Inc., Irvine, CA or EnCore Enspire Breast Biopsy System, C.R. Bard Inc., Tempe, AZ, USA).

### Surgical excision

The subsequent surgical excision was carried out in all analysed cases. For each patient, both tumour and all planned incisions were marked on the skin before surgery. Preoperative skin markings were done with the patient in the upright position. In non-palpable tumours, needle localisation of the lesion was made under the local anaesthesia. Needle localisation was performed using Accura BLN device (MDTech, Gainesville, FL, USA; distribution in Europe by PBN Medicals, Stenlose, Denmark) through the post-biopsy scar. After the needle was placed and its position was confirmed, the hook wire was deployed and the ultrasound view was obtained to confirm wire position. In all the cases, postoperative tissue specimen was oriented in three dimensions by the surgeon and inked to assist in identifying margins.

### Statistical analysis

Data were collected in a prospective manner and then were entered into computer database. In each case, patient age, history of breast cancer, radiological presentation, pathologic findings, clinical character and mass size (the longest diameter in ultrasound examination) were measured. Mean, standard deviation, median and range values were calculated when appropriate. Rates of upstaging to IDC were calculated and compared to clinical character and size of DCIS. Continuous variables were compared between two groups using Mann–Whitney U-test. Categorical variables were analysed using Pearson’s chi-square test. *P* values less than 0.05 were considered statistically significant.

## Results

Median age of patients (mean, SD, range) was 63 years (61.3, 11.5, 27–88). Median diameter of DCIS was 11 mm (13.9, 9.4, 6–60). Foci of IDC were found in postoperative specimen in 56 % of DCIS presenting as a sonographic mass (35/63). Patient age did not differ significantly between groups with and without final IDC (median, mean, SD): 63, 61.4, 12.5 vs 62, 61.2, 10.6 years, respectively (*P* = 0.659). The difference of DCIS size between these groups was statistically important (median, mean, SD): 13, 17.3, 11.4 vs 9.5, 9.8, 3.2 mm, respectively (*P* = 0.0003). Palpable lesions were significantly more often upstaged to invasive cancer: 76 vs 38 % (*P* = 0.0027). Rates of underestimation were associated with the size of DCIS: 91 % (10/11) for greater than 20 mm, 64 % (14/22) for 10–20 mm and 37 % (11/30) for equal or smaller than 10 mm. Association between the risk of upstaging to invasive disease and clinical character as well as diameter of DCIS using different cut-off points (10, 15, 20 and 30 mm; *P* = 0.0040, 0.0003, 0.9717 and 0.0371, respectively) is presented in Table 1.

**Table 1:** Rate of upstaging to invasive ductal cancer after subsequent surgical excision

DCIS presenting as a mass		Rate of upstaging	*P* value
All mass DCIS		56 % (35/63)	-
Palpability	Absent	38 % (13/34)	<0.01
	Present	76 % (22/29)	
Size	≤10 mm	37 % (11/30)	<0.01
	>10 mm	73 % (24/33)	
	≤15 mm	43 % (20/47)	<0.001
	>15 mm	94 % (15/16)	
	≤20 mm	48 % (25/52)	>0.05
	>20 mm	91 % (10/11)	
	≤30 mm	52 % (30/58)	<0.05
	>30 mm	100 % (5/5)	

## Discussion

Based on the final pathology, we found the upstaging of DCIS to IDC in 56 % of patients, which is higher than 18–43 % reported in current literature [6, 12–16]. Rate of upstaging depends on the biopsy method (core-needle vs vacuum-assisted) [12–15] as well as radiological guidance (ultrasound-guided vs stereotactic) and average tumour size [16]. High rate in our group, despite minimum five tissue cores and the use of vacuum-assisted technique (high-volume specimens) in non-palpable DCIS, cannot be simply explained by these factors.

It is rather due to the fact that all investigated DCIS was presenting as a mass on mammography, which is related to important risk of upstaging [14, 16]. Moreover, some studies report that sonographic mass (as in our study) can be a significant [6] and independent [13] predictor of invasion. This kind of radiological presentation can reflect higher potential of local invasiveness: breaking through the basement membrane of the breast duct and infiltration of deeper tissues. It is still a subject of debate if the clinical character of mass (palpable vs non-palpable) plays an important role: some data are against [12], some are for [15]. Current multivariate analyses revealed statistically significant association between palpability of DCIS and the risk of invasion (46–70 % vs 23–30 %) [13, 14]. In a series of Schulz and colleagues, it was the essential and only independent factor [6]. Nevertheless, in the absence of properly randomized studies, the true correlation remains unclear and significant and significant conclusions cannot be drawn.

Traditionally, DCIS with the extent over 4–5 cm has been considered a high-risk lesion for upstaging to invasive cancer [5]. These patients should undergo an axillary nodal staging with sentinel node biopsy. However, during the few last years, the recommended cut-off point much decreased in size, to 25 mm [7]. Some recent studies report 20 mm as a significant value [14, 15, 17]. Lee and colleagues and Park and co-investigators suggest even smaller cut-off point: 15 mm (34.6 vs 0.4 %) and 10 mm (60.3 vs 39.7 %), respectively [12, 13]. Data from this last report are in concordance with our findings that in some clinical subtypes of DCIS with specific and less common radiographic manifestation as a mass, the risk of upstaging to IDC after subsequent surgical excision is remarkably high even when the lesion is very small.

Our study has some limitations. First, this is a single centre series—our findings may not be applicable to specific patient cohort managed in other institution. Second, the lack of control group (e.g. DCIS without mass) hinders the ability to prove statistical significance. Third, a variety of minimally invasive biopsy techniques (vacuum assisted and core needle with automated gun) and needle gauges were used. The use of one biopsy technique with one needle gauge would be ideal for the confidence of results. Despite these important limitations, our findings are potentially interesting from a clinical point of view.

## Conclusions

Considerable risk of DCIS upstaging in patients with even small lesions presenting as a mass can be helpful for optimal treatment planning. It should be taken into account in decision-making process with regard to the extent of surgical intervention.
